# Supplementary material for: Single-Cell Transcriptomics Reveals the Complexity of the Tumor Microenvironment of Treatment-Naive Osteosarcoma
Source: Front Oncol. 2021 Jul 21;11:709210. doi: 10.3389/fonc.2021.709210 (PMC8335545; doi:10.3389/fonc.2021.709210)
Supplement: Supplementary Document 1 — Kaplan-Meier curve of the 518 genes associated with survival. [file DataSheet_1.zip › Supplementary Document 1/sur.TP53I11.pdf]

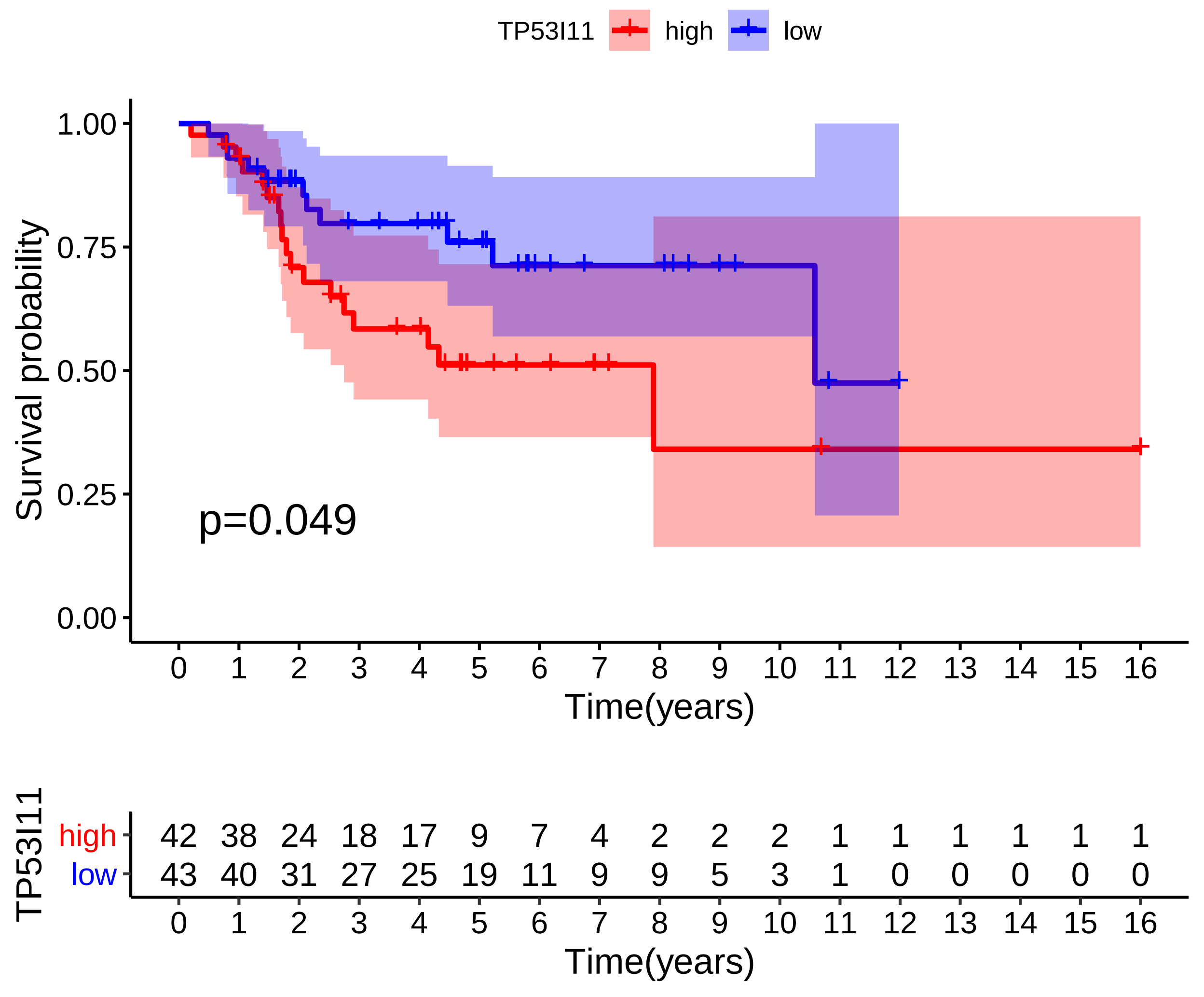
TP53I11
high
low
Survival probability
1.00
0.75
0.50
0.25
0.00
p=0.049
0 1 2 3 4 5 6 7 8 9 10 11 12 13 14 15 16
Time(years)
TP53I11
high 42 38 24 18 17 9 7 4 2 2 2 1 1 1 1 1 1
low 43 40 31 27 25 19 11 9 9 5 3 1 0 0 0 0 0
0 1 2 3 4 5 6 7 8 9 10 11 12 13 14 15 16
Time(years)
